# Structome Analysis of Virulent *Mycobacterium tuberculosis*, Which Survives with Only 700 Ribosomes per 0.1 fl of Cytoplasm

**DOI:** 10.1371/journal.pone.0117109

**Published:** 2015-01-28

**Authors:** Hiroyuki Yamada, Masashi Yamaguchi, Kinuyo Chikamatsu, Akio Aono, Satoshi Mitarai

**Affiliations:** 1 Department of Mycobacterium Reference and Research, the Research Institute of Tuberculosis, Japan Anti-Tuberculosis Association, Kiyose, Tokyo, Japan; 2 Medical Mycology Research Center, Chiba University, Chiba, Japan; Federal University of Rio de Janeiro, BRAZIL

## Abstract

We previously reported the exquisite preservation of the ultrastructures of virulent *Mycobacterium tuberculosis* cells processed through cryofixation and rapid freeze substitution. Here, we report the “structome” analysis (i.e., the quantitative three-dimensional structural analysis of a whole cell at the electron microscopic level) of virulent *M. tuberculosis* using serial ultrathin sections prepared after cryofixation and rapid freeze substitution and analyzed by transmission electron microscopy. Five *M. tuberculosis* cells, which were contained in the serial ultrathin cross sections encompassing from one end to the other, were cut into 24, 36, 69, 55, and 63 serial ultrathin sections, respectively. On average, the cells were 2.71 ± 1.05 μm in length, and the average diameter of the cell was 0.345 ± 0.029 μm. The outer membrane and plasma membrane surface areas were 3.04 ± 1.33 μm^2^ and 2.67 ± 1.19 μm^2^, respectively. The cell, outer membrane, periplasm, plasma membrane, and cytoplasm volumes were 0.293 ± 0.113 fl (= μm^3^), 0.006 ± 0.003 fl, 0.060 ± 0.021 fl, 0.019 ± 0.008 fl, and 0.210 ± 0.091 fl, respectively. The average total ribosome number was 1,672 ± 568, and the ribosome density was 716.5 ± 171.4/0.1 fl. This is the first report of a structome analysis of *M. tuberculosis* cells prepared as serial ultrathin sections following cryofixation and rapid freeze substitution and examined by transmission electron microscopy. These data are based on the direct measurement and enumeration of exquisitely preserved single-cell structures in transmission electron microscopy images rather than calculations or assumptions from indirect biochemical or molecular biological data. In addition, these data may explain the slow growth of *M. tuberculosis* and enhance understanding of the structural properties related to the expression of antigenicity, acid-fastness, and the mechanism of drug resistance, particularly in regard to the ratio of target to drug concentrations.

## Introduction

Bacteria can be observed with the naked eye as turbidity in a liquid medium or as colonies on the surface of a solid medium. Under light or fluorescent microscopic analysis, bacteria appear as stained or fluorescence-emitting small round cocci or rod-shaped bacilli. These macroscopic and microscopic observations are equivalent to observing the biosphere from an aircraft flying at high altitude or observing large animals or tall plants on the earth’s surface from an aircraft at low altitude. Although such observations provide surficial and numerical information useful for scientific and clinical investigations, they reveal only limited superficial information regarding individual bacterial cells. Biological phenomena occur in the cell envelope and cytoplasm of bacteria; therefore, we must utilize electron microscopy, particularly transmission electron microscopy (TEM), to observe the ultrastructure of bacteria in detail. Although TEM examinations of bacteria provide a variety of information, in general these examinations are highly qualitative because the intact cytoplasmic ultrastructure is poorly preserved by conventional chemical fixation, which makes it difficult to perform quantitative analyses.

Recent reports have revealed that cryofixation (CRF) and rapid freeze substitution (RFS) provide exquisite preservation of whole yeast and bacterial cells [1–8]. The authors of these studies examined various cellular properties and components using CRF-RFS–processed epoxy resin–embedded samples. Yamaguchi *et al*. determined the number and volume of organelles, including the mitochondria, endoplasmic reticulum, Golgi apparatus, and ribosomes using serial ultrathin sections of yeast cells. They proposed the term “structome” for this technique, which is defined as the ‘quantitative and three-dimensional structural information of a whole cell at the electron microscopic level’ [3, 5].

*Mycobacterium tuberculosis*, which is the causative agent of tuberculosis, contains a single, circular, double-stranded DNA molecule consisting of approximately 4.3 million base pairs, located in the cytoplasm [9]. Several investigators have calculated and estimated the number of mycobacterial cell components, including ribosomes, based on molecular biological and biochemical evidence [10–14].

Here, we report the results of a structome analysis of individual *M*. *tuberculosis* cells. *M*. *tuberculosis* cells were preserved by CRF-RFS and prepared for examination as serial ultrathin sections of epoxy resin–embedded cells. Cellular properties, including size, surface area, and volume, were directly measured based on electron micrographs, and the ribosomes scattered throughout the cytoplasm were enumerated. The ribosome density (number per 0.1 fl of cytoplasm) for *M*. *tuberculosis* was calculated and compared to that determined for yeast cells [2, 5] as well as previously published estimations [10–14]. This is the first report of a structome analysis of individual *M*. *tuberculosis* cells using serial ultrathin sections and direct enumeration. These data will aid future investigations of the fundamental cell biology, pathogenesis, and drug resistance of *M*. *tuberculosis*.

## Materials and Methods

### Bacteria

*M*. *tuberculosis* H37Rv (ATCC 27294) was cultured in 50 ml of Middlebrook 7H9 (Becton Dickinson, Sparks, MD, USA) supplemented with albumin (Fraction V), dextrose, and catalase enrichment (Becton Dickinson) and 0.05% Tween 80 contained in a 125-ml Erlenmeyer flask with a plain bottom (Nalgene, 4112–0125, NY, USA) for 2 weeks. Exponentially growing cells were used. Aliquots (1 ml) of cultured cells were transferred to sterile microcentrifuge tubes and centrifuged at 10,000 × *g* for 1 min. Usually, we used 6 ml of cultured cell suspension. The supernatants were discarded, and the remaining pellets were collected in two microcentrifuge tubes.

### CRF-RFS and epoxy resin embedding

The sandwich method was performed as described previously [2–7]. Briefly, a portion (<1 μl) of the highly concentrated bacterial pellet prepared as described above was applied to a glow-discharge-treated single-hole cupper grid (Veco; hole size, 0.1-mm diameter) and then sandwiched with another glow-discharge-treated single-hole grid. The grids were then picked up with tweezers and frozen by plunging them into melting propane (propane cooled with liquid nitrogen in a cooling device) for 20 seconds, as described previously [5]. The pair of grids was transferred, detached in liquid nitrogen, and immersed quickly into 2% osmium tetroxide/acetone solution and then placed in the device described above and cooled. Next, the samples were transferred from the bio-safety facility and placed in a freezer at −85°C for several days, after which they were allowed to come to room temperature over several days in a conventional area of the laboratory. Then, the osmium tetroxide/acetone solution was discarded and the samples were washed with absolute acetone three times at room temperature. The samples were then embedded in Spurr’s resin using Leica/LKB Embedding Capsule Easy Molds (8-mm diameter) and polymerized at 70°C for 16 hours.

### Preparation of serial ultrathin sections and TEM methods

More than 500 serial ultrathin sections with an average thickness of 55 nm were cut with an Ultracut E ultramicrotome (Reichert-Jung Co., Wien, Austria) equipped with a diamond knife and then picked up using single-slot cupper grids with a slot size of 2.0 × 1.0 mm (Maxtaform HF49, Tonbridge, UK) on 12 single-hole grids. Serial ultrathin sections were then transferred onto formvar support film mounted on an aluminum rack with 20 pores of 4-mm diameter [4]. The serial ultrathin sections were then dried, detached from the rack with support of the formvar, and stained with uranyl acetate and lead citrate. Of the 12 grids prepared, the serial ultrathin sections on 5 grids were damaged; therefore, the serial ultrathin sections on the remaining 7 grids were subjected to TEM examination. Among these 7 grids, serial ultrathin sections containing 5 cells on 5 grids were examined by TEM.

TEM examinations were performed using a JEOL JEM-1230 electron microscope operated at 80 kV. At the beginning of the examination, printed images of micrographs collected at low magnification (×2,000 to ×2,500) were searched for longitudinal cell profiles in the serial ultrathin sections in order to complete the examination at high magnification with a small number of serial ultrathin sections. However, because cell contents often dropped out from sections, especially those near the edge of the cell, serial cross sections encompassing the cell from one end to the other were searched at low magnification. Next, 5 cells in a total of 140 serial ultrathin sections were examined at higher magnification (×30,000, ×60,000, or ×80,000).

### Image analysis and ribosome enumeration

Images obtained from negative scanning through Adobe Photoshop Elements (version 9) with CanoScan 8800F were saved as TIFF files and analyzed using ImageJ and Fiji software [15, 16]. Briefly, cell length was calculated by multiplying the number by 55 nm (representing the thickness of each section). The diameter (minor and major axes), perimeter, and thickness of the plasma membrane (PM), outer membrane (OM), and cell envelope of each cell were measured as a pixel value using the line selection menu in the ImageJ/Fiji window as well as a scale bar recorded on the same negative. Measured pixel values were converted to μm or nm according to the measured pixel value of the scale bar on the corresponding negatives.

The cross-sectional area of each cell was determined using the ‘Measure’ command in the ‘Analyze’ menu of ImageJ/Fiji by tracing the OM using the polygonal selection menu in the ImageJ window and converting the area result above into μm^2^ by multiplying the square of the ratio of scale (nm) on the scanned negative by its pixel value. The cross-sectional area of each cell’s cytoplasm was determined by tracing the PM in a like manner. The OM and PM surface areas (μm^2^) were calculated as the cumulative area of a trapezium of the cell in each section using the formula for calculating the area of a trapezoid, where the perimeter of the OM and PM in a given section and the previous section were used as the upper base and lower base, respectively, and the section thickness (0.055 μm [55 nm]) was used as the height.

The volume (fl, = μm^3^) of each cell was calculated as the cumulative volume of cylinders having the cell’s cross-sectional area as the base and the section thickness (0.055 μm [55 nm]) as the height. The volumes of the OM and PM were calculated by multiplying the surface area of each membrane by its thickness (0.002 μm and 0.007 μm, respectively). The volume of the periplasm was calculated by subtracting the cytoplasmic and OM volumes from the cell volume.

Ribosomes as electron dense particles with 10 ~ 20 nm diameter in the cytoplasm of the cell cross-section in each serial ultrathin section were enumerated using the ‘Multi-point Tool’ in ImageJ/ Fiji [15, 16]. The total number of ribosomes in each cell and the number of ribosomes per 0.1 fl of cytoplasm were calculated based on the volume of each cell determined as described above.

### Statistics

Averages and standard deviations for the diameter, area of the cross section in each serial ultrathin section, surface areas of the OM and PM, and cell volume were calculated for each cell and compared.

## Results and Discussion

We previously reported the excellent preservation of the ultrastructure of *M*. *tuberculosis* cells provided by CRF-RFS [5, 7, 8]. CRF-RFS preserves not only the cell envelope with OM but also ribosomes within the cytoplasm.

**Figure 1:**
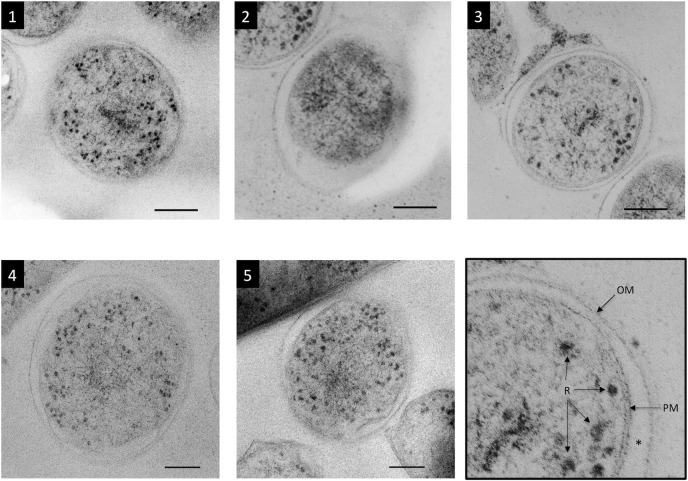
TEM images of five cross-sectioned *M*. *tuberculosis* cells. Cells were cut in the middle. The outer membrane (OM), periplasm (asterisk), plasma membrane (PM), and ribosomes (R) are visible as shown in the bottom right panel (enlarged image of cell 3). The cytoplasm of cell 2 appeared to have degraded, as evidenced by its dark color and fewer ribosomes. Cell 3 can be seen to the upper left of cell 2. Bar: 100 nm.

In this study, more than 500 serial ultrathin sections were prepared from CRF-RFS samples, and five *M*. *tuberculosis* cells contained in the serial ultrathin sections were examined (Fig. 1, S1–S5 Figs.). Cell structures were measured and the number of ribosomes contained in the cytoplasm was determined. This is the first report of a structome analysis of individual *M*. *tuberculosis* cells, and there are no other reports describing the examination of bacteria in serial ultrathin sections prepared from epoxy resin–embedded CRF-RFS–preserved samples.

### One-dimensional analysis

The length and diameter of five *M*. *tuberculosis* cells were measured using ImageJ/Fiji software, as described in the Materials and Methods section. Table 1 lists the results of a one-dimensional structome analysis of the five cells. Cells 1, 2, 3, 4, and 5 were cut into 24, 36, 69, 55, and 63 serial ultrathin sections, respectively. Cells 1, 2, 3, 4, and 5 were 1.32, 1.93, 3.80, 3.03, and 3.47 μm in length, respectively, for an average cell length of 2.71 ± 1.05 μm. As shown in S2 and S3 Figs., the serial ultrathin sections containing cells 2 and 3 were not continuous due to changing of the grid. That is, several serial ultrathin sections between numbers 30 and 31 for cell 2 and between numbers 67 and 68 for cell 3 may have been dropped out or mounted on the metal frame of the next grid (S2 and S3 Figs.). Therefore, these two cells may be slightly longer than the data. In addition, because cell length was determined based on the thickness of each serial ultrathin section and the number of serial ultrathin sections covering the cell, curved cells may be slightly longer than the calculated data indicate. Furthermore, because of examination protocol on serial ultrathin sections, whole cell profiles encompassing from one end to other can be observed in the shorter cells than the longer.

**Table 1:** One-dimensional profiles of *M*. *tuberculosis* cells.

Cell	Length (μm)	Average Diameter (μm) (Cell)	Average Diameter (μm) (Cytoplasm)	Aspect Ratio
(Number of serial ultrathin sections) ^1^				
Cell 1 (24)	1.32	0.366	0.326	3.61
Cell 2 (35)	1.93	0.320	0.278	6.01
Cell 3 (69)	3.80	0.293	0.273	13.0
Cell 4 (55)	3.03	0.351	0.306	8.63
Cell 5 (67)	3.47	0.348	0.299	9.96
Average	2.71	0.345	0.297	8.23
SD	1.05	0.029	0.022	3.60

^1^ Values in parenthesis indicate the number of serial ultrathin sections required to complete whole cell profile measurement.

**Figure 2:**
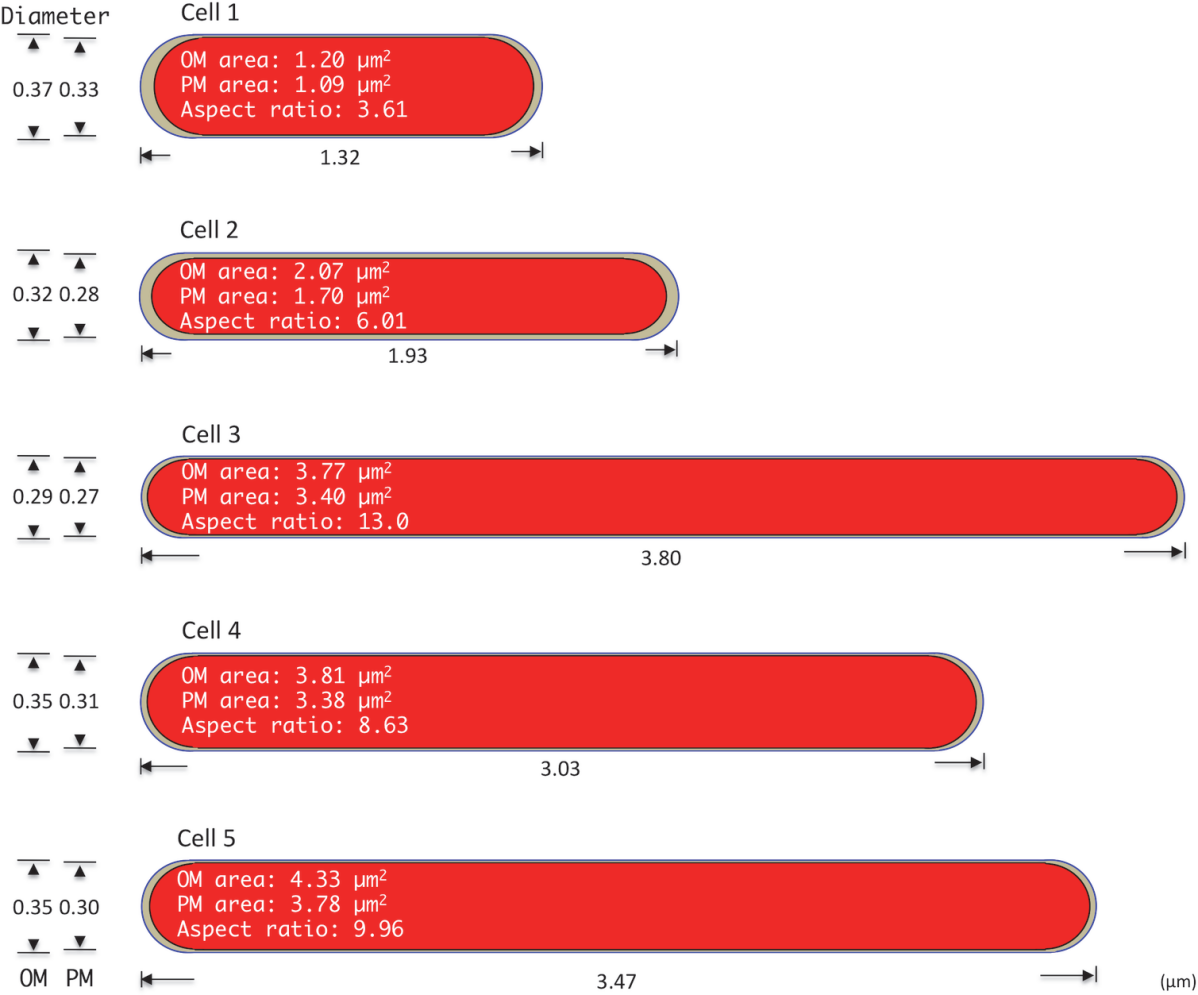
One- and two-dimensional properties of five *M*. *tuberculosis* cells obtained from serial ultrathin section TEM examination. The size of each schema correlates with the cell’s relative width and length. The diameter, length, aspect ratio, outer membrane (OM) surface area, and plasma membrane (PM) surface area of each of five cells are indicated.

Cell diameter values were very similar, with average OM and PM diameters of 0.345 ± 0.029 μm (range, 0.293–0.366 μm) and 0.297 ± 0.022 μm (range, 0.273–0.326 μm), respectively. The average aspect ratio was 8.23 ± 3.60 (Table 1 and Fig. 2). Because the lengths of cells 3, 4, and 5 were 3-fold greater than that of the smallest cell (cell 1), it is suggested that cell division may occur independently of the length of the parent cell.

### Two-dimensional analysis

Two-dimensional profiles for the five *M*. *tuberculosis* cells were calculated from values measured using ImageJ/Fiji software, as described in the Materials and Methods section. Table 2 lists the average cross-sectional area of each whole cell (outlined by the OM), its cytoplasm (outlined by the PM), and the surface areas of the OM and PM. The average cross-sectional and cytoplasmic areas were 0.116 ± 0.028 μm^2^ (range, 0.079–0.147 μm^2^) and 0.090 ± 0.026 μm^2^ (range, 0.057–0.116 μm^2^), respectively (Fig. 2), and the average OM and PM surface areas were 3.037 ± 1.333 μm^2^ (range, 1.201–4.328 μm^2^) and 2.672 ± 1.1911 μm^2^ (range, 1.095–3.775 μm^2^), respectively. Because the surface areas of cells 3, 4, and 5 were 3-fold greater than that of the smallest cell (cell 1), it is suggested that cell division may occur independently of the surface area of the parent cell.

**Table 2:** Two-dimensional profiles of *M*. *tuberculosis* cells.

Cell	Area (μm^2^)			
(Number of serial ultrathin sections) ^1^	Average Cross-section (Cell)	Average Cross-section (Cytoplasm)	Outer Membrane Surface	Plasma Membrane Surface
Cell 1 (24)	0.134	0.108	1.201	1.095
Cell 2 (35)	0.096	0.057	2.072	1.704
Cell 3 (69)	0.079	0.066	3.773	3.402
Cell 4 (55)	0.124	0.102	3.809	3.383
Cell 5 (67)	0.147	0.116	4.328	3.775
Average	0.116	0.090	3.037	2.672
SD	0.028	0.026	1.333	1.191

^1^ Values in parenthesis indicate the number of serial ultrathin sections required to complete whole cell profile measurement.

### Three-dimensional (3D) analysis

**Figure 3:**
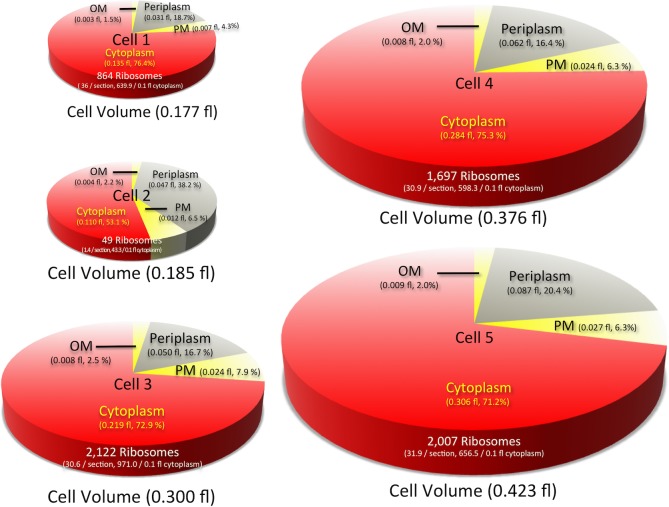
Pie graphs showing the volumes of the cell compartments for each of the five *M*. *tuberculosis* cells examined. The size of each pie correlates with the cell’s relative volume. The whole-cell volume, proportion of the volumes of the cell compartments, total number of ribosomes, and ribosome density for each of five cells are indicated.

The volume of each of the five *M*. *tuberculosis* cells was calculated using measurements taken with ImageJ/Fiji software, as described in the Materials and Methods section. Data regarding the 3D profile of each cell are shown in Table 3 and Fig. 3. The average whole-cell (as outlined by the OM) and cytoplasmic (as outlined by the PM) volumes were 0.293 ± 0.113 fl (range, 0.177–0.429 fl) and 0.210 ± 0.091 fl (range, 0.110–0.306 fl), respectively. In addition, the average OM, PM, and periplasmic volumes were 0.006 ± 0.003 fl (range, 0.002–0.009 fl), 0.019 ± 0.008 fl (range, 0.008–0.027 fl), and 0.060 ± 0.021 fl (range, 0.031–0.088 fl), respectively. Because the volumes of cells 3, 4, and 5 were 2-fold greater than that of the smallest cell (cell 1), it is suggested that cell division may occur independently of the volume of the parent cell.

**Table 3:** Three-dimensional profiles of *M*. *tuberculosis* cells.

Cell	Volume (fl)				
(Number of serial ultrathin sections) ^1^	Cell	Outer Membrane	Periplasm	Plasma Membrane	Cytoplasm
Cell 1 (24)	0.177	0.002	0.031	0.008	0.135
Cell 2 (35)	0.185	0.004	0.075	0.012	0.110
Cell 3 (69)	0.300	0.008	0.050	0.024	0.218
Cell 4 (55)	0.376	0.008	0.062	0.024	0.284
Cell 5 (67)	0.429	0.009	0.088	0.027	0.306
Average	0.293	0.006	0.060	0.019	0.210
SD	0.113	0.003	0.021	0.008	0.091

^1^ Values in parenthesis indicate the number of serial ultrathin sections required to complete whole cell profile measurement.

### Ribosome enumeration

The number of ribosomes in the cytoplasm of each cell was also determined (Table 4 and Fig. 3). This is the first report of the direct whole-cell enumeration of bacterial ribosomes in the cytoplasm based on TEM examinations of serial ultrathin sections images, as there are no previously published reports describing examinations of serial ultrathin sections of bacteria preserved by CRF-RFS. The average total ribosome number, average number per section, and average number per 0.1 fl of cytoplasm for the five cells examined in this study were 1,347 ± 877 (range, 49–2,122), 26.0 ± 18.6 (range, 1.4–36), and 583.2 ± 333 (range, 43.3–971), respectively.

**Table 4:** Ribosome enumeration.

Cell	Average per section (range)	Density per 0.1 fl	Total
(Number of serial ultrathin sections) ^1^			
Cell 1 (24)	36.0	640.2	864
	(0–94)		
Cell 2 (35)	1.4	43.3	49
	(0–7)		
Cell 3 (69)	29.8	971.0	2,122
	(0–70)		
Cell4 (55)	30.9	598.3	1,697
	(0–89)		
Cel5 (67)	31.9	656.5	2,007
	(0–72)		
Average	32.2 [26.0]^2^	716.5 [583.2]^2^	1,672 [1,347]^2^
	(0–94)		
SD	3.7 [18.6]^2^	171.4 [333.0]^2^	568 [877]^2^

^1^ Values in parenthesis indicate the number of serial ultrathin sections required to complete whole cell profile measurement.

^2^ Values between square brackets are the calculation results including the value of cell 2.

Cell 2 contained only 49 ribosomes in the entire cell (not just in a single section), whereas each of the other 4 cells contained more than 850 ribosomes, and 3 of these cells (cells 3, 4, and 5) contained more than 1,600 ribosomes each. Some of the serial ultrathin sections of the cytoplasm of cell 2 showed even or scanty profiles, whereas other serial ultrathin sections were more electron dense (S2 Fig.). These profiles suggest that cell 2 was dead before RFS treatment and that degradation of the ribosomes had begun. This decomposition of cell 2 may be thought of as an artifact of preparation resulting from CRF-RFS processing. However, it is highly unlikely that only cell 2 had begun to decompose due to processing because cells 2 and 3 coexisted in the same sections, as shown in Fig. 1, S2 and S3 Figs., and all cells, including cells 2 and 3, were prepared using the same procedure. In addition, the volume of the periplasmic space for cell 2 was relatively large (Table 3, Fig. 1 and S2 Fig.), a characteristic thought to be indicative of cytoplasm degradation. This suggests that cell 2 was already dead at the start of the CRF-RFS procedure. Excluding the data for cell 2, the average ribosome number per cell and the average ribosome number per 0.1 fl of cytoplasm for the remaining 4 cells were 1,672 and 716.5, respectively.

The number of ribosomes per *M*. *bovis* BCG cell has been estimated or calculated in several studies, with results ranging from 690 to 4,400 [10–13]. The calculated values were based on the proportion of rRNA in total RNA and the amount of ribosome-associated nucleotides [12]. Estimated numbers were based on the assumption that all of the transcribed rRNA molecules are used for the assembly of functional ribosomes with subunits of ribosome proteins, with no consideration given to rRNA molecules being derived from dead cells in the sample. In addition, because the ribosome numbers were estimated in bulk without measuring the volume of individual cells, the ribosome density could not be determined. Therefore, differences in ribosome enumerations based on estimates and direct observations suggest the possible presence of free rRNAs and free subunits of ribosomal proteins, which cannot be observed through TEM because these free molecules do not localize as high-electron-density particles. These observations and interpretations indicate that determinations of rRNA copy number may lead to overestimation of the number of functional ribosomes and that precise enumeration of functional ribosomes requires direct observation of properly fixed and exquisitely preserved cytoplasm using TEM.

### Comparison of ribosome density between *M*. *tuberculosis* and yeast

To date, there are only a few reports of quantitative structome analyses of microorganisms using TEM, including structome analyses of the yeast *Exophiala dermatitidis* and *Saccharomyces cerevisiae* [2, 6]. These studies reported values for the average number of ribosomes per 0.1 fl of 1,100 (11,000/fl) and 1,950 (19,500/fl) for *E*. *dermatitidis* and *S*. *cerevisiae*, respectively. In the present study, we found an average density of ribosomes in *M*. *tuberculosis* cells of approximately 720/0.1 fl, a density that is about one-half and one-third that of *E*. *dermatitidis* and *S*. *cerevisiae*, respectively (Table 5). The reported ribosome densities for *E*. *dermatitidis* and *S*. *cerevisiae* [2, 6] may be underestimates, as those studies enumerated ribosomes only in the cytoplasm and not in the nucleus, which comprises 7–10% of the cell volume. In addition, the study of *S*. *cerevisiae* compared structome data obtained in analyses of cells in both early G1 and late G1 phase. Other than a significantly higher number of autophagosomes in the cells in early G1 phase compared with late G1 phase, no other significant differences were observed, although the authors mentioned that the higher autophagosome number in early G1 phase cells may not be truly significant because the *P-value* was close to 5% [6]. These results suggest that there may be no significant differences in the ribosome density in *M*. *tuberculosis* cells between stationary and exponential phases, where it is difficult to determine whether a single *M*. *tuberculosis* cell within a population is in exponential phase or stationary phase, even with TEM.

**Table 5:** Comparison of ribosome number and density between *M*. *tuberculosis* and yeast cells.

Organism	Density per 0.1 fl	Average total ribosomes per cell (range)
(Number of examined cell)	(range)	
*M*. *tuberculosis* (5)	716.5 [597.9]^1^	1,672 [1,347]^1^
	(640 ~ 971)	(864 ~ 2,122)
*E*. *dermatitidis* (5)^2^	1,100	195,000
	(970 ~ 1,340)	(112,000 ~ 336,000)
*S*. *cerevisiae* (6)^3^	1,950	195,000
	(1,770 ~ 2,060)	(115,000 ~ 272,000)

^1^ Values between square brackets are the calculation results including the value of cell 2.

^2^ Data described in reference 2.

^3^ Data described in reference 6.

### Ribosome density–based drug targeting

With respect to ribosome-targeting drugs used in tuberculosis chemotherapy, few studies have examined the relationship between the number of target molecules in a single *M*. *tuberculosis* cell and the drug concentration in the environment surrounding the cell. For example, most cases of streptomycin (STR; C_21_H_39_N_7_O_12_; MW, 581.57 Da) resistance are attributed to mutations within either *rrs* in 16S rRNA, *rpsL* (encoding ribosomal protein S12), or *gidB* (encoding 7-methylguanosine [m^7^G], a methyltransferase specific for rRNA) [17–19]. However, some STR-resistant strains harbor none of the above-mentioned mutations [20]. In this study, the average ribosome density was about 720 per 0.1 fl of cytoplasm, as calculated based on direct enumeration using serial ultrathin sections. In an environment containing STR at a concentration of 1.0 μg/ml (as is used in MGIT 960 drug susceptibility tests [21]), there would be about 100 STR molecules/0.1 fl. If the STR molecules could freely penetrate into the cytoplasm of *M*. *tuberculosis* cells and the concentration inside the cell is equal to that of the surrounding environment, the drug molecules will attack only one-seventh of ribosomes at any given point in time. It is not known how many direct binding events between ribosomes and STR molecules are required to kill a single bacterial cell. Therefore, because some cases of STR resistance cannot be explained by mutations in the target molecules, it is critical that the relationship between drug resistance and the drug to target molecule ratio be considered, along with how many drug molecules are required to kill a single bacterial cell and how many targets within a single bacterial cell must be attacked to effect killing.

### Significance of structome analysis of serial ultrathin sections

In general, most microbiological studies are performed on clumps of bacterial cells or bulk bacterial populations, both of which may contain a large number of dead cells. The results of such studies are always expressed as average values, and these values may be under- or overestimations of the true values. In contrast, microscopy allows for direct observation of the dynamics of single cells with temporal or spatial continuity, enabling researchers to distinguish between living and dead cells. It is generally believed that because they share identical genetic information, all cells in a population of bacteria derived from a single strain should show the same phenotype, whether they are part of a colony or in suspension in liquid medium. However, it was recently reported that major differences in cell shape and susceptibility to antibiotics may exist between isogenic cells in a colony originating from a single cell [22–25]. Our structome analyses show that the size properties and ribosome density may vary from cell to cell, even though the cells may be of the same strain. Time-lapse microscopy experiments and TEM structome analyses of serial ultrathin sections of single cells containing the same genomic information have revealed that individual cells have both similarities and differences, as is the case with human identical twins, whose fingerprints normally differ. This is the simplest demonstration that genes are not omnipotent [25]; that is, the final phenotype is not determined solely by genetic information. Therefore, we must observe living or properly fixed cells using suitable microscopy methods in order to understand the phenomena occurring in individual bacterial cells.

As mentioned in a previous report [6], 3D structural analyses are now possible thanks to newly developed techniques and devices, including electron tomography [26], cryo-electron microscopy with vitreous sections [26, 27], serial block-face–scanning electron microscopy [28], and focused ion beam–scanning electron microscopy [29]. These new techniques allow 3D reconstruction of protein molecules, microorganisms, organelles, and cells automatically in less time than conventional analyses using ultrathin sections and TEM. However, these techniques do have some drawbacks, including the possibility of blind angles in tomography, very low contrast in cryo-electron microscopy with vitreous sections, and degradation of cell components such as ribosomes as a result of treating specimens with reagents such as potassium ferric cyanide to enhance membrane contrast in focused ion beam–scanning electron microscopy and serial block-face scanning electron microscopy. Therefore, although these techniques have made 3D structural analyses easier to perform, the analyses themselves are exclusively qualitative rather than quantitative, and the resolution of the images obtained using these procedures is no better than that of conventional approaches involving TEM analysis of serial ultrathin sections. Nevertheless, future improvements in sample preparation and resolution in these new methods should overcome the above-mentioned problems and provide new approaches for structome analysis.

## Conclusions

This is the first report of a TEM structome analysis of *M*. *tuberculosis* cells in serial ultrathin sections, based on direct measurement and enumeration of exquisitely preserved structures in living single cells rather than calculations or assumptions based on indirect biochemical or molecular biological data. Our data will enhance other investigations in fundamental biology, immunogenicity, and the pathogenicity of *M*. *tuberculosis*, as well as vaccine development, and mechanisms of drug resistance because our data about the surface area and the volume of PM and OM can provide insights into the capacity of these structures to contain various molecules, which may elicit immunogenicity and/or pathogenicity or may act as drug-target. In addition, our results will enhance quantitative analyses of drug-target interactions, particularly with respect to the drug to target molecule ratio of ribosome-targeting drugs.

## Supporting Information

S1 Fig Serial ultrathin section array of cell 1. A total of 24 serial ultrathin sections were cut to a thickness of 55 nm.
(TIF)

S2 Fig Serial ultrathin section array of cell 2. A total of 36 serial ultrathin sections were cut to a thickness of 55 nm. Cell 3 is co-localized in the cross sections, as seen from top-left to bottom-left throughout the array. The cell profile ended at the 36^th^ ultrathin section.
(TIFF)

S3 Fig Serial ultrathin section array of cell 3. A total of 69 serial ultrathin sections were cut to a thickness of 55 nm. The cell profile begins in the 2^nd^ ultrathin section and ends at the 70^th^ ultrathin section.
(TIFF)

S4 Fig Serial ultrathin section array of cell 4. A total of 55 serial ultrathin sections were cut to a thickness of 55 nm. The cell profile begins in the 1^st^ ultrathin section and ends at the 55^th^ ultrathin section.
(TIFF)

S5 Fig Serial ultrathin section array of cell 5. A total of 63 serial ultrathin sections were cut to a thickness of 55 nm. The cell profile begins in the 2^nd^ ultrathin section.
(TIFF)

S1 Table Dimensional properties determined from serial ultrathin sections of cell 1. (XLSX)

S2 Table Dimensional properties determined from serial ultrathin sections of cell 2. (XLSX)

S3 Table Dimensional properties determined from serial ultrathin sections of cell 3. (XLSX)

S4 Table Dimensional properties determined from serial ultrathin sections of cell 4. (XLSX)

S5 Table Dimensional properties determined from serial ultrathin sections of cell 5. (XLSX)

## References

[1] Yamaguchi M, Biswas SK, Suzuki Y, Furukawa H, Sameshima M, et al (2002) The spindle pole body duplicates in early G1 phase in a pathogenic yeast *Exophiala dermatitidis*: an ultrastructural study. Exp Cell Res 279: 71–79. 12213215 10.1006/excr.2002.5578

[2] Biswas SK, Yamaguchi M, Naoe N, Takashima T, Takeo K (2003) Quantitative three-dimensional structural analysis of *Exophiala dermatitidis* yeast cells by freeze-substitution and serial ultrathin sectioning. J Electron Microsc 52: 133–143. 12868584 10.1093/jmicro/52.2.133

[3] Yamaguchi M (2006) Structome of *Exophiala* yeast cells determined by freeze-substitution and serial ultrathin sectioning electron microscopy. Current Trends Microbiol 2: 1–12.

[4] Yamaguchi M, Okada H, Namiki Y (2009) Smart specimen preparation for freeze substitution and serial ultrathin sectioning of yeast cells. J Electron Microsc 58: 261–266. 10.1093/jmicro/dfp013 19289851

[5] Yamada H, Mitarai S, Chikamatsu K, Mizuno K, Yamaguchi M (2010) Novel freeze-substitution electron microscopy provides new aspects of virulent *Mycobacterium tuberculosis* with visualization of the outer membrane and satisfying biosafety requirements. J Microbiol Methods 80: 14–18. 10.1016/j.mimet.2009.09.022 19799941

[6] Yamaguchi M, Namiki Y, Okada H, Mori Y, Furukawa H (2011) Structome of *Saccharomyces cerevisiae* determined by freeze-substitution and serial ultrathin-sectioning electron microscopy. J Electron Microsc 60: 321–335. 10.1093/jmicro/dfr052 21908548

[7] Yamada H, Bhatt A, Danev R, Fujiwara N, Maeda S, et al (2012) Non-acid-fastness in *Mycobacterium tuberculosis ∆kasB* mutant correlates with the cell envelope electron density. Tuberculosis 92: 351–357. 10.1016/j.tube.2012.02.006 22516756

[8] Yamada H, Chikamatsu K, Aono A, Mitarai S (2014) Pre-fixation of virulent *Mycobacterium tuberculosis* with glutaraldehyde preserves exquisite ultrastructure on transmission electron microscopy through cryofixation and freeze-substitution with osmium-acetone at ultralow temperature. J Microbiol Methods 96: 50–55. 10.1016/j.mimet.2013.10.019 24200708

[9] Cole ST, Brosch R, Parkhill J, Garnier T, Churcher C, et al (1998) Deciphering the biology of *Mycobacterium tuberculosis* from the complete genome sequence. Nature 393: 537–544. 9634230 10.1038/31159

[10] Cox RA (2003) Correlation of the rate of protein synthesis and the third power of the RNA: protein ratio in *Escherichia coli* and *Mycobacterium tuberculosis* . Microbiology 149: 729–737. 12634341 10.1099/mic.0.25645-0

[11] Cox RA (2003) Quantitative relationships for specific growth rates and macromolecular compositions of *Mycobacterium tuberculosis*, *Streptomyces coelicolor* A3(2) and *Escherichia coli* B/r: an integrative theoretical approach. Microbiology 150: 1413–1426. 10.1099/mic.0.26560-0 15133103

[12] Beste DJ, Peters J, Hooper T, Avignone-Rossa C, Bushell ME, et al (2005) Compiling a molecular inventory for *Mycobacterium bovis* BCG at two growth rates: evidence for growth rate-mediated regulation of ribosome biosynthesis and lipid metabolism. J Bacteriol 187: 1677–1684. 15716438 10.1128/JB.187.5.1677-1684.2005 PMC1064002

[13] Cox RA (2007) A scheme for the analysis of microarray measurements based on a quantitative theoretical framework for bacterial cell growth: application to studies of *Mycobacterium tuberculosis* . Microbiology 153: 3337–3349. 17906133 10.1099/mic.0.2007/005868-0

[14] Cox RA, Garcia MJ (2013) Adaptation of mycobacteria to growth conditions: a theoretical analysis of changes in gene expression revealed by microarrays. PLoS One 8: e59883 10.1371/journal.pone.0059883 23593152 PMC3625197

[15] Rasband WS (1997–2014) ImageJ. U S National Institutes of Health, Bethesda, Maryland, USA, http://imagej.nih.gov/ij/.

[16] Schindelin J, Arganda-Carreras I, Frise E, Kaynig V, Longair M, et al (2012) Fiji: an open-source platform for biological-image analysis. Nat Methods 9: 676–682. 10.1038/nmeth.2019 22743772 PMC3855844

[17] Pelchovich G, Zhuravlev A, Gophna U (2013) Effect of ribosome-targeting antibiotics on streptomycin-resistant *Mycobacterium* mutants in the *rpsL* gene. Int J Antimicrob Agents 42: 129–132. 10.1016/j.ijantimicag.2013.04.001 23664678

[18] Tekwu EM, Sidze LK, Assam JP, Tedom JC, Tchatchouang S, et al (2014) Sequence analysis for detection of drug resistance in *Mycobacterium tuberculosis* complex isolates from the Central Region of Cameroon. BMC Microbiol 14: 113 10.1186/1471-2180-14-113 24884632 PMC4017682

[19] Okamoto S, Tamaru A, Nakajima C, Nishimura K, Tanaka Y, et al (2007) Loss of a conserved 7-methylguanosine modification in 16S rRNA confers low-level streptomycin resistance in bacteria. Mol Microbiol 63: 1096–1106. 17238915 10.1111/j.1365-2958.2006.05585.x

[20] Jagielski T, Ignatowska H, Bakula Z, Dziewit L, Napiorkowska A, et al (2014) Screening for streptomycin resistance-conferring mutations in *Mycobacterium tuberculosis* clinical isolates from Poland. PLoS One 9: e100078 10.1371/journal.pone.0100078 24937123 PMC4061058

[21] Ardito F, Posteraro B, Sanguinetti M, Zanetti S, Fadda G (2001) Evaluation of BACTEC Mycobacteria Growth Indicator Tube (MGIT 960) automated system for drug susceptibility testing of *Mycobacterium tuberculosis* . J Clin Microbiol 39: 4440–4444. 11724858 10.1128/JCM.39.12.4440-4444.2001 PMC88562

[22] Aldridge BB, Fernandez-Suarez M, Heller D, Ambravaneswaran V, Irimia D, et al (2012) Asymmetry and aging of mycobacterial cells lead to variable growth and antibiotic susceptibility. Science 335: 100–104. 10.1126/science.1216166 22174129 PMC3397429

[23] Wakamoto Y, Dhar N, Chait R, Schneider K, Signorino-Gelo F, et al (2013) Dynamic persistence of antibiotic-stressed mycobacteria. Science 339: 91–95. 10.1126/science.1229858 23288538

[24] Santi I, Dhar N, Bousbaine D, Wakamoto Y, McKinney JD (2013) Single-cell dynamics of the chromosome replication and cell division cycles in mycobacteria. Nat Commun 4: 2470 10.1038/ncomms3470 24036848

[25] Calder N (1976) The human conspiracy. London: British Broadcasting Corporation p75.

[26] Hoffmann C, Leis A, Niederweis M, Plitzko JM, Engelhardt H (2008) Disclosure of the mycobacterial outer membrane: cryo-electron tomography and vitreous sections reveal the lipid bilayer structure. Proc Natl Acad Sci U S A 105; 3963–3967. 10.1073/pnas.0709530105 18316738 PMC2268800

[27] Zuber B, Chami M, Houssin C, Dubochet J, Griffiths G, et al (2008) Direct visualization of the outer membrane of mycobacteria and corynebacteria in their native state. J Bacteriol 190; 5672–5680. 10.1128/JB.01919-07 18567661 PMC2519390

[28] Denk W, Horstmann H (2004) Serial block-face scanning electron microscopy to reconstruct three-dimensional tissue nanostructure. PLoS Biol 2; e329 15514700 10.1371/journal.pbio.0020329 PMC524270

[29] Ohta K, Sadayama S, Togo A, Higashi R, Tanoue R, et al (2012) Beam deceleration for block-face scanning electron microscopy of embedded biological tissue. Micron 43; 612–620. 10.1016/j.micron.2011.11.001 22285616

