# Supplementary figures and images for: Structome Analysis of Virulent Mycobacterium tuberculosis, Which Survives with Only 700 Ribosomes per 0.1 fl of Cytoplasm
Source: PLoS One. 2015 Jan 28;10(1):e0117109. doi: 10.1371/journal.pone.0117109 (PMC4309607; doi:10.1371/journal.pone.0117109)

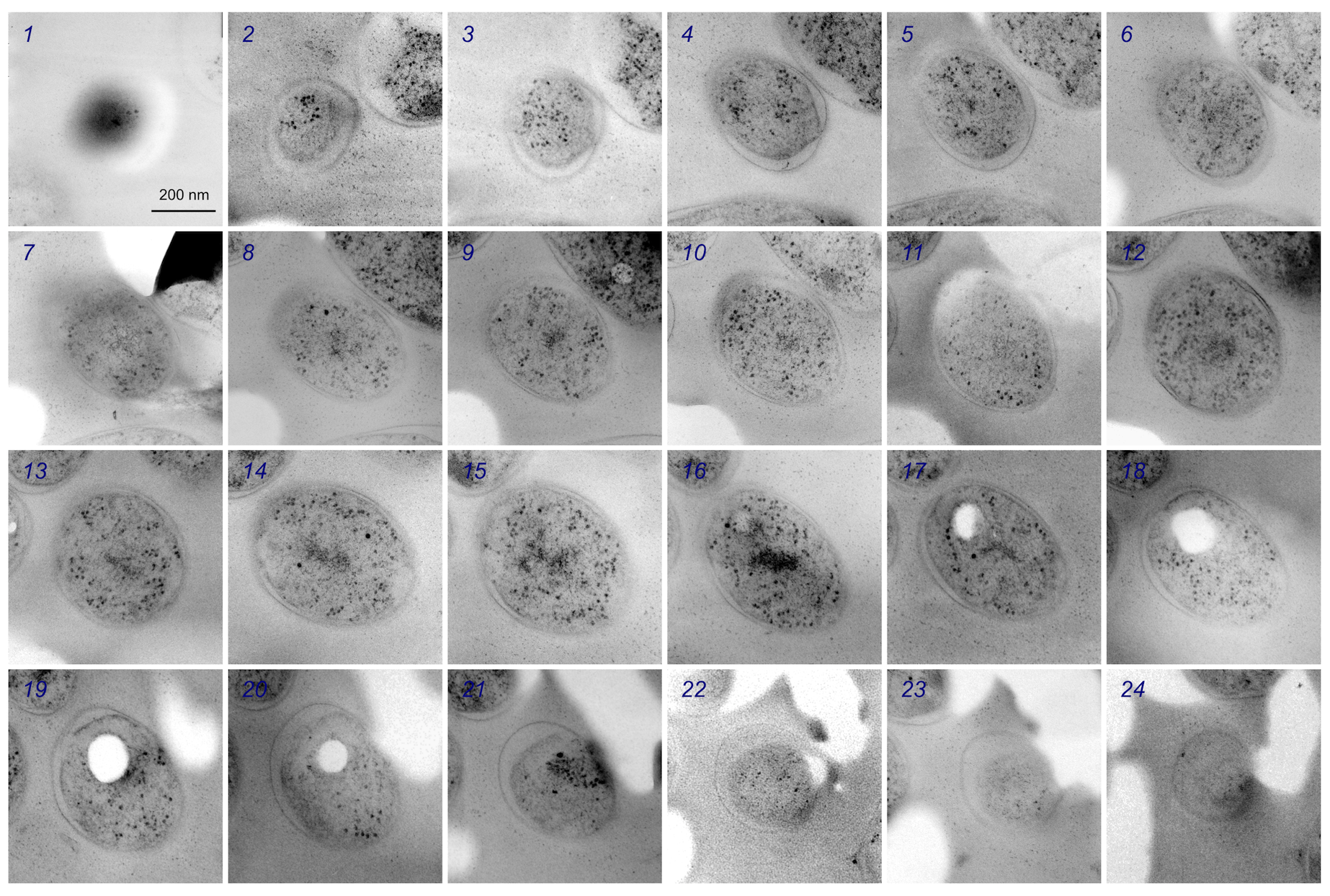

Supplement: S1 Fig — A total of 24 serial ultrathin sections were cut to a thickness of 55 nm. (TIF) [file pone.0117109.s001.tif]

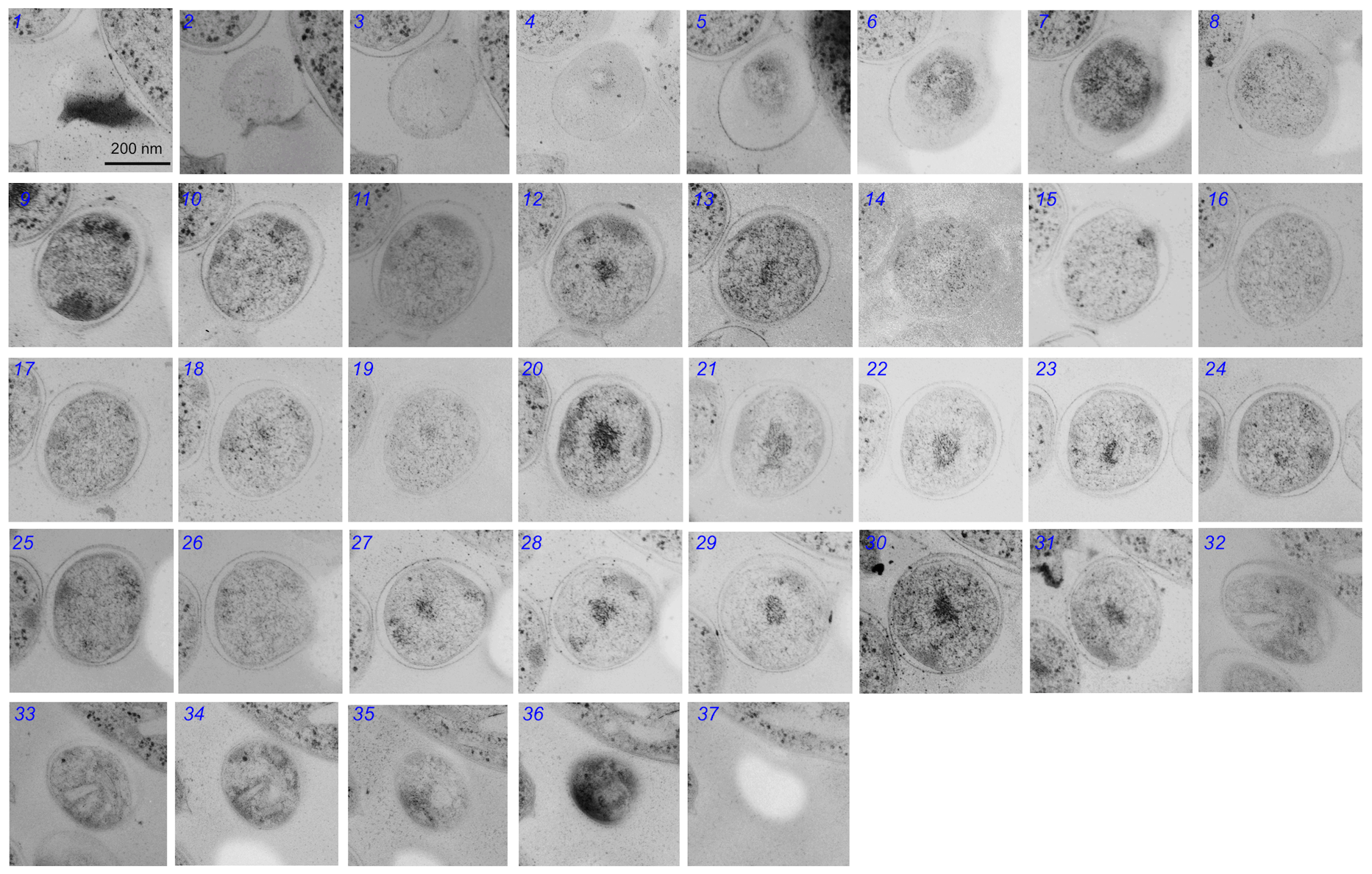

Supplement: S2 Fig — A total of 36 serial ultrathin sections were cut to a thickness of 55 nm. Cell 3 is co-localized in the cross sections, as seen from top-left to bottom-left throughout the array. The cell profile ended at the 36th ultrathin section. (TIFF) [file pone.0117109.s002.tiff]

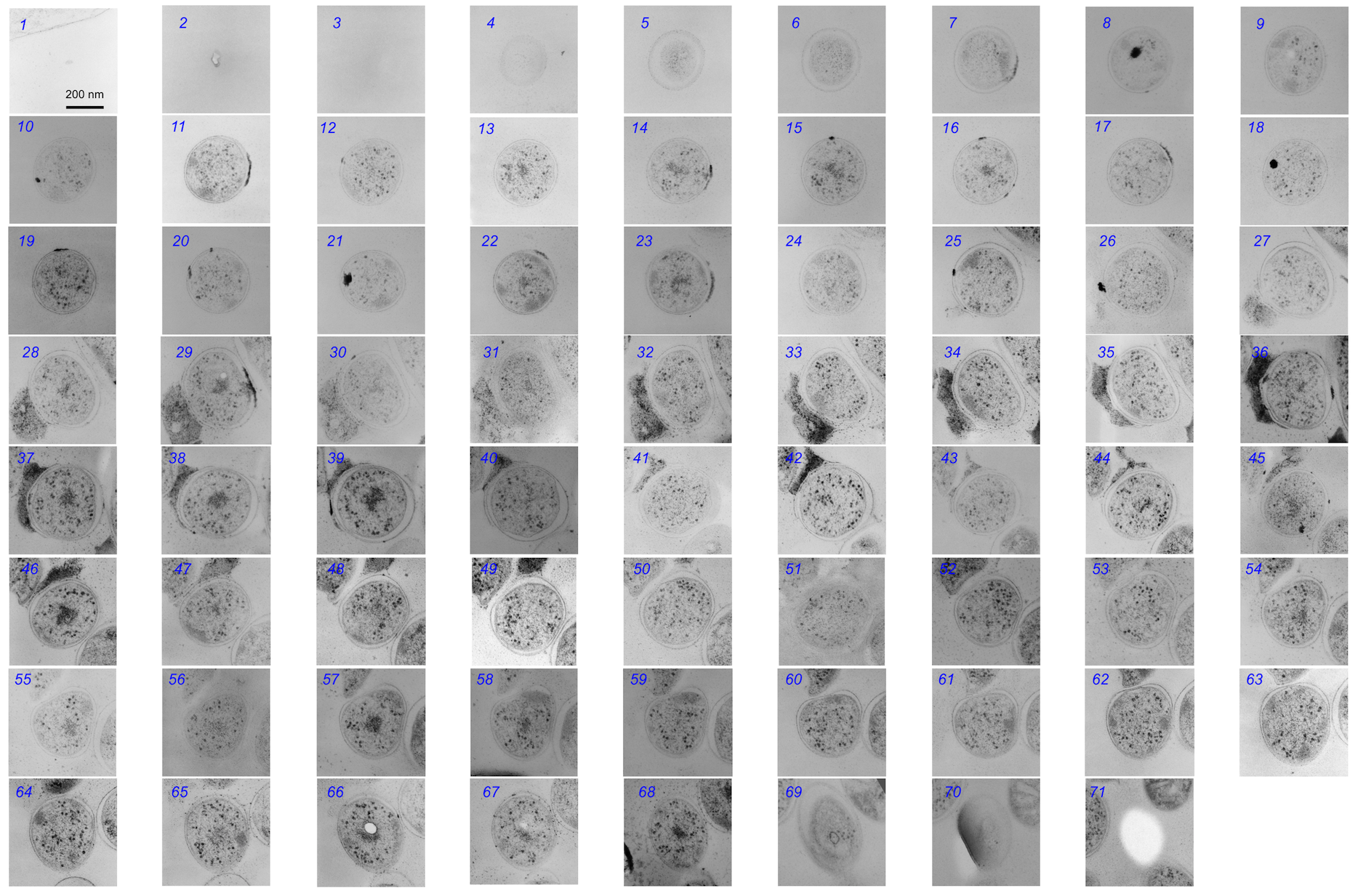

Supplement: S3 Fig — A total of 69 serial ultrathin sections were cut to a thickness of 55 nm. The cell profile begins in the 2nd ultrathin section and ends at the 70th ultrathin section. (TIFF) [file pone.0117109.s003.tiff]

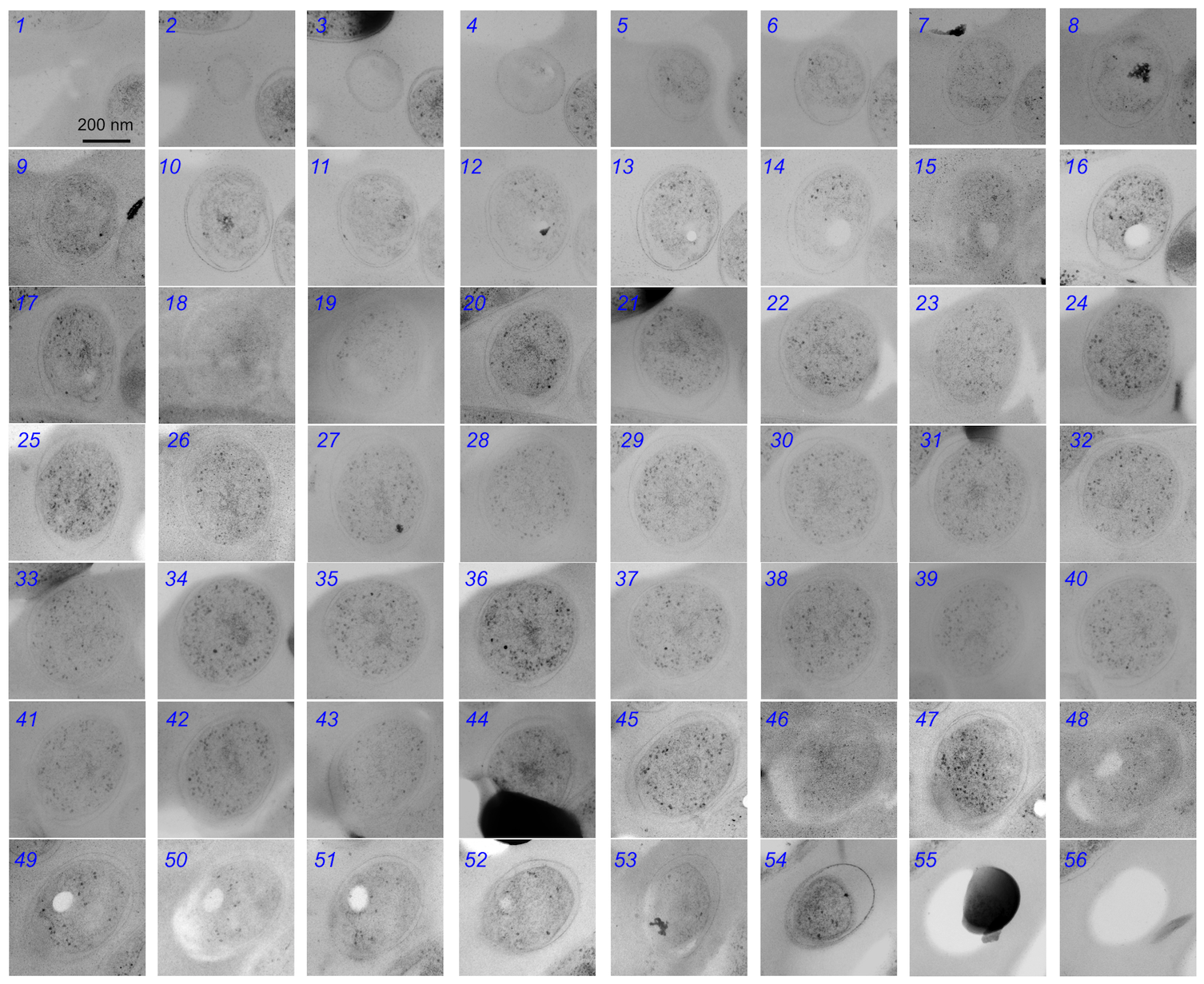

Supplement: S4 Fig — A total of 55 serial ultrathin sections were cut to a thickness of 55 nm. The cell profile begins in the 1st ultrathin section and ends at the 55th ultrathin section. (TIFF) [file pone.0117109.s004.tiff]

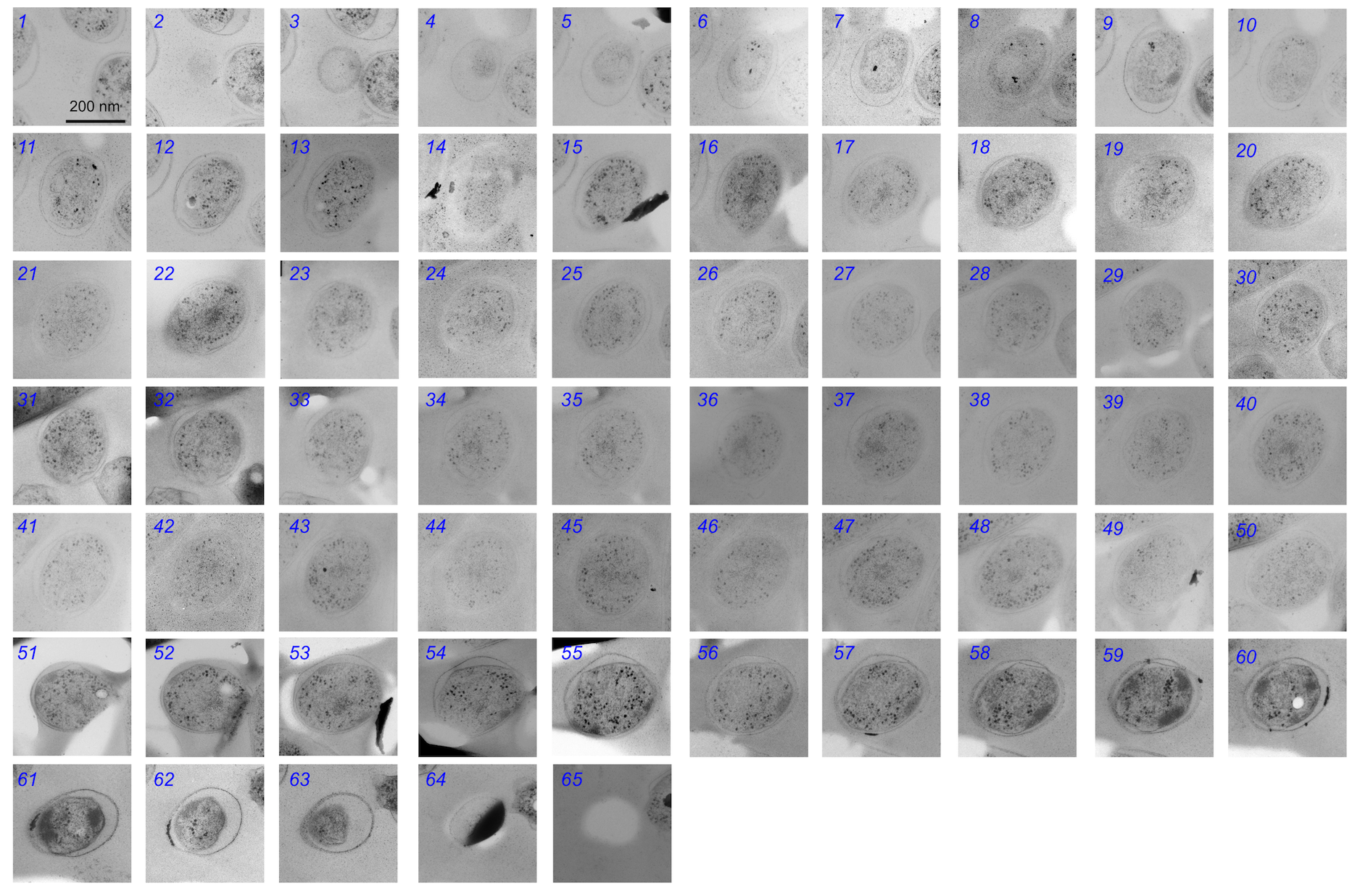

Supplement: S5 Fig — A total of 63 serial ultrathin sections were cut to a thickness of 55 nm. The cell profile begins in the 2nd ultrathin section. (TIFF) [file pone.0117109.s005.tiff]
